# Remarks of J. McF. Gaston, M. D, on Taking the Chair of Surgery in the Southern Medical College, Atlanta, October 8, 1884

**Published:** 1884-11-20

**Authors:** 


### Southern Medical Record.

EDITORS:

THOMAS S. POWELL, M.D. R. C. WORD, M.D.

*R. C. WORD, M.D., Managing Editor.*

All Communications and Letters on Business connected with the RECORD must be addressed to the Managing Editor.

VOL. XIV. ATLANTA, GA., NOVEMBER 20, 1884. No. 11.

### ORIGINAL AND SELECTED ARTICLES.

### REMARKS OF J. McF. GASTON, M. D., ON TAKING THE CHAIR OF SURGERY IN THE SOUTHERN MEDICAL COLLEGE, ATLANTA, OCTOBER 8, 1884.

[With a few words of commendation Dr. J. Thad. Johnson presented his successor.

After some preliminary remarks touching his appreciation of the honor of his appointment, and of its responsibilities, and referring to his surgical experience in the Confederate Army, and in Brazil, to which country he repaired for a time after the war, Professor Gaston remarked as follows :]

A distinguished scientist asserts that if the mountains and vallies on the face of the earth, and the depressions and elevations in the bed of the ocean, over the entire face of the globe, were levelled by cutting down the high lands and filling up the excavations, the water which is now banked in by the land would spread out over the broad expanse of the earth, so as to envelop its spherical outline with a superimposed sea of great depth.

This type of compensation in the correction of all the departures from a smooth plain, that the scattered waters may be reunited and invest the whole globe, affords a fit illustration of the world of thought. Should all the irregularities and deviations from the proper standard in philosophy, science, and arts be corrected and levelled down we might hope to see all overlaid with the panoply of truth. While some are building up, others are tearing down the temple of Science ; but when we cast a glance over the wide range of intellect, it will be seen that the hills and hollows of false theory and hurtful practices in the various departments of our profession are being reduced to correct principles, so that error must eventually disappear, and truth will prevail.

Each of us should consider himself as an instrument in bringing about the triumph of Truth in all that pertains to the progress of the medical profession, and every one should contribute toward this great result. Some can accomplish more than others, yet zeal and perseverance will ensure success for all.

An individual under the influence of some mental hallucination was observed to move his jaws as in the act of mastication, and to keep up this chewing operation unremittingly. Upon seeking an explanation of this incessant movement of his jaws it was ascertained that he had taken up the impression that the entire outer crust of the earth was undergoing a progressive combustion, and that the herculean task was imposed upon him of masticating a paste which was to be applied by piecemeal along the vast border of this slow fire ! He labored assiduously to fulfill this stupendious task, and presented an example worthy of imitation by the medical profession in attempting to avert the progress of Disease, which is corroding, gradually but surely, the health of mankind in all parts of the habitable globe. An African surgeon is reported as covering the incision after performing the Cæsarian operation with a paste chewed by those around the patient !

Notwithstanding the combined efforts of those who are going forth, annually, from our schools to join the great multitudes of experienced men who are chewing and applying their paste to this great phagedenic ulcer, it is still sapping the vitality of the human race, and we have the cry resounding over the hills and plains of this vast continent : Come to the rescue !

Demand creates supply, and, as a consequence, large numbers of medical colleges have sprung up throughout the United States, and a large number of medical journals are extending a knowledge of the healing art, while many books have been published ; so that the resources of this country, in the medical and surgical fields are unequalled to-day by those enjoyed in any other.

In addition to the other available means of improvement in knowledge, polyclinic schools for practitioners have been inaugurated in several of our large cities ; and this seems to me as the greatest element of progress in medical education that marks the present era of advancement in progressive matters. It indicates that medical men are alive to the fact that education does not cease with getting their diplomas; and some of us who have been engaged in practice for a longer period than most of you have sojourned on the earth still require to learn. The great school of Experience is opening up new points for investigation, and we must continue to study, if we would profit by the grand researches of the patient experimenter in the field of science of the present day.

When we consider the important advances made in these latter years in abdominal and thoracic surgery, it may be reasonably inferred that similar developments may reward the researches in other branches of surgery; and the field to be explored is so vast that all may find ample employment in carrying out the investigations which have been so auspiciously undertaken by specialists in the various departments of practice.

If medical men generally could be taught enough to know their own ignorance, and have it brought home to them that they need to learn much in keeping up with the march of science, an important step would have been gained towards reaching the high standard of education to be aimed at by physicians.

There are divers fields of observation which have yielded important results; and while the experiments of the laboratory give us favorable indications in physiology and pathology, it is through clinical observations that our therapeutic agencies have become developed.

No mere theory or hypothesis is any longer received by medical men without the proof of facts, and the accurate records of the experience of large numbers of practitioners in the different phases of diseases have proven highly advantageous in reaching correct conclusions in regard to treatment.

Since the magnificent achievement of Jenner in protecting the human race from the ravages of small pox by vaccination, nothing has been done in the prophylactic department which compares with the attainments of Pasteur in protecting animals from splenic fever and mankind from that destructive pestilence known as charbon by the French, and designated malignant pustule amongst us. If the bright star of promise which has risen over hydrophobia does not set in darkness this *savant* will have attained a glorious distinction, unequaled by any other benefactor of his fellow beings.

While the investigations of Koch have attracted much attention throughout the scientific world, there is not as yet any definitely established practical issue of his researches; and many of the propositions announced by him require to be demonstrated by results before they can be accepted as facts. The crucial test of utility needs to be applied in the outgrowth of his experiments. While Koch claims to have demonstrated that the comma-bacillus is the source of cholera, he has likewise, according to Prof. Hugo Engel, "discovered that the becteria of putrefaction are inimical to, and capable of destroying, the comma-bacillus, and in this connection it was noted that whenever the sewers in Toulon were cleansed or disinfected the cholera became worse, which fact would tend to explain the reported immunity from the disease among the scavengers of Toulon and Naples." If this view be correct, all our preconceived ideas of hygiene are upset in regard to the conditions which predispose to the propagation of cholera, and the threatened cities should offer a premium on filth, instead of adopting strenuous measures for securing cleanliness.

It has recently been communicated, also, that "Dr. Bossana, of Pharo Hospital, at Marseilles, telegraphed on September 9 that Drs. Reitsel and Ricoli had just informed him that several animals which they had inoculated with Dr. Koch's microbes had died with choleraic symptoms—results which Dr. Koch had failed to obtain."

The probability, however, is that the fate of the comma-bacillus will in the end be like that of the tubercle microbes, which gained so much notoriety for this theorist and experimenter, and yet has thus far brought no important practical results in the management of the disease. Thus far the investigations of Koch have not secured the benefits to mankind which have followed the cautious and painstaking researches of Pasteur.

I would present the claims of Surgery to your special attention, but not in the light of a distinct branch of practice, as it is only regarded in the curriculum of the college as a preparatory step for entering upon the duties of a general practitioner.

There are no schools in this country for the special training of surgeons, as there are in Great Britain ; and while the taste and capacity of some may fit them better for this department than for the other duties of the medical profession, it is not desirable that any one shall begin the study of medicine with the intention of devoting himself to a specialty. All should prepare themselves with due care for any and every service that may be required of them, and should a peculiar adaptation for some branch of practice be developed by experience, such an one may perfect himself in its details and enter this field of duty. I desire to impress upon every student of medicine the importance of diligent attention to the instructions received in the several departments of the prescribed course, and yet it would give me great satisfaction to observe the greatest zeal in the devotion of every member of the class to a thorough understanding of Surgery. Your careful attention and studious consideration are therefore expected for my future teachings in this department, which I will strive to render worthy of your adoption in practice.

As the best plan to learn anything thoroughly is to teach it to others, I realize that the School of Surgery lies open to me as well as to you, and it is hoped that we may reap its advantages.

The key-stone of the arch of Surgery is Anatomy, and this bears the same relation to physiology that mathematics does to physics. It is a fixed quantity, which does not vary in the normal condition of the organization, and should be mastered by all who would make progress in surgery, as a knowledge of the relations of different organs and of histology is essential to the proper performance of the most simple surgical operation.

A knowledge of the other departments, though quite distinct, is requisite for the preliminary and subsequent management of operations ; and materia medica, with theraputics, and the treatment of incidental diseases, belong to the surgeon's domain, while chemistry assists in diagnosis.

It would seem that the process of natural labor might be ignored by surgeons, yet changes often take place in the course of uncomplicated delivery which require surgical interference, and it behooves all to know what is normal and what is abnormal in this process. As to the array of prophylactics for the proper discharge of this natural function of woman we may say that they are more honored in the breach than by their observance, and Nature may be allowed to take her own course until signs of trouble are presented. But your Professor of Obstetrics will advise you to be always on the lookout, and to meet any impending difficulty promptly.

No true surgeon is a mere operator, but must take an enlarged view of the physical state of his patient, and act always understandingly and advisedly for warding off the dangers to life.

It becomes us, then, to inquire at the outset of our course what belongs to the surgical department.

The literal signification of the term Surgery is hand-work ; but while operative surgery implies manual or instrumental appliances in the treatment of the case, the more extended sphere of the principles and practice of surgery embodies all the physical disorders of structure, with their relations to the general organization, and the means of their correction.

A proper definition of surgery covers all the ostensible departures from a healthy standard, and may be considered as the science of perceptible abnormal developments, whether external or internal. A distinction based upon the location of diseases, exteriorly or interiorly, has been recognised by some as designating the cases pertaining to surgery, and the practice of medicine, respectively ; but this classification has no proper foundation, since many deep-seated diseases belong to the domain of the surgeon, while some external affections fall in the department of the medical practioner. I would, therefore, ignore this mode of localizing the physical troubles which may be treated surgically, and designate all abnormal modifications of structure, wherever found, with the measures of correction, as the appropriate field of surgery. While surgical disorders are generally limited to circumscribed areas, the means of relief are not confined to local appliances, and the treatment may extend to agencies which operate upon the entire organism ; so that surgery affects not only special regions, but has other remote and general influences upon the whole body.

It will be understood by the extension of surgical disorders from a local modification to a general influence, that while some cases require only topical measures of treatment others call for constitutional remedies, and, accordingly, the scope of surgical practice cannot be limited to operations and manipulations, or local applications of whatever kind they may be. An illustration of this point is afforded in (syphilis) an affection which presents local lesions that demand attention, yet the underlying element of the disease, which is by far the most urgent feature to be combatted, is the constitutional taint, and the surgeon's province extends to the eradication of the general disease in like manner as in making appropriate topical applications for the local manifestations.

The same principle holds in regard to all surgical cases in which the general system is involved by the progress of the disease, or in which constitutional disturbance results from any operative procedure, and the surgeon must of necessity take into account this phase of surgical practice. The preparation of a patient to undergo an operation is often of more importance than the proper execution of the cutting, and the regimen and dressings, subsequently, have much to do with the result. It makes the difference between failure and success to give proper attention to the minor steps of an operation ; and I recall a case of amputation of the leg in which the ligation of the arteries was entrusted to another person, who did it so unskillfully as to allow one of the ligatures to escape after a few hours, and but for a faithful nurse death would have ensued.

In the matter of ligatures, the constriction may be too great and lead to a division of the tissues, as in a case (of ablation of the uterus) under my observation in Brazil, where my companion, in compliance with a suggestion from me, tied the pedicle in three divisions, but the ligatures were drawn so tightly as to cut the tissues, and in less than half an hour after the final dressings were concluded secondary hemorhage occurred, that led to the death of the patient within five days. The postmortem revealed the fact that the division of blood-vessels from the cutting by the ligatures had caused the bleeding, and hence we see the great importance attached to the proper execution of the minor details of an operation for securing a salutary issue.

It is known that the most successful operators for abdominal tumors are especially careful in all the minutiæ, and upon this hinges the recovery of the patient.

If I could impress upon all who have to treat surgical cases the importance of little matters, a great point would be gained towards success in operations ; and it is well to proceed in any case requiring cutting instruments as if expecting that trouble may follow, so as to induce caution in every step of the operation, and in the subsequent dressings. No forecast can in every case anticipate what may occur, but when everything is done which prudence dictates the surgeon has no cause for any reflections upon himself as to the consequences, and such unforeseen accidents belong to the class of contingencies which cannot be obviated.

Originality of conception and promptness in execution should characterise the action of the surgeon under emergencies ; and there are few cases of operations upon deepseated parts which do not require some modifications of the programme in the progressive steps, so that new developments must be met upon the spur of the moment with appropriate measures. The most simple case—as that in which a diagnosis of a fatty tumor over the pectoral muscle, leads to an incision of six or eight inches, which is carried down to the muscular tissues without encountering any abnormal development—demands all the surgical tact of the operator to determine immediately what course to pursue. Not finding the tumor on the outer surface of the muscle, the indurated mass is still perceived and supposed to be an adenoid tumor beneath the tissues of the muscle, so that the appropriate dissection is made to pass beneath the lower border of the pectoralis-major, and when the facia are all divided, a soft, fluctuating mass is discerned in contact with the ribs and the intercostal muscles. Here a new doubt comes up to the mind of the operator, as to the possibility of aneurism of the subclavian or axillary artery, and must be solved before another step is taken. A little coolness, and cautious examination, satisfies him there is no pulsation, and leads to the conclusion that it is a purulent collection, which is soon discharged and all embarrassment ended. I cite this case, which has actually occurred under my own observation, in this city, as an illustration of the importance of tact on the part of the surgeon under emergencies.

While urging the use of every precaution against unfavorable results in operations I would inculcate boldness in undertaking that which offers a fair prospect of relief, and in cases of inevitable death from the operation of disease a resort to heroic measures is justifiable as a last recourse. Get the acquiescence, if possible, of the patient and friends in what is requisite for the case, and never allow the consideration of the opinions of the outside world to interfere with the performance of a grave and responsible operation which may possibly save the life of a patient, otherwise doomed to death. Should we hesitate in cutting the cord upon finding a man suspended at a great height by a rope about his neck lest he may be hurt in the fall? Certainly not; and no more is it questionable as to life-and-death operations.

Surgery becomes dangerous only in the hands of the ignorant or unskillful operator, and woe be to him who lays hold of the surgeon's knife without proper intelligence to guide it, or without the courage to accomplish what is necessary for the safety of his patient!

The qualification for practicing surgery does not consist only in thoroughly comprehending the different steps to be taken in performing an operation, but in having the nerve and self-possession which will enable him to go forward without embarrassment with all its details, so that the intellectual, emotional, and physical constitution must be fitly associated to constitute a good operator. Those who do not possess these requisites had better leave surgical cases to the care of others.

The elements of common sense enter to a greater extent into American surgery than is found in other regions of the world, and, instead of those artificial scientific divisions which serve the ends of book-making, we find such classifications recognized as promote a better understanding of the nature of cases. This simplyfing of terms goes so far as to recognize railroad surgery, clyclone surgery, etc., which convey an impression of the character of the injuries to be treated without giving any distinction of the parts or regions involved. In all such results of violence shock is quite a prominent characteristic, and must be taken into the account for a successful management of the case.

Precedents have not the same significance in this country as in the older and more staid portions of the world, where a "thus saith the record" is the warrant for proceeding. In a country like England, whose men do not wear trowsers because their fathers dispensed with this nether garment, and where railroad lines do not check baggage because their stage coaches of olden time found it unnecssary for travelers, it is not surprising that surgeons should be wedded to old modes of practicing surgery, as a legitimate succession of the prerogative of the barbers! In the same spirit that they hold to the divine right of kings, they adhere to prescribed forms of operating, and shut their eyes to many advances and surgical triumphs, as "operative audacities."

The principles and practice of Hunter, Abernethy, Lister, Bell, Cooper, and Brodie made a notable departure from their predecessors, and material advances also have been made by the surgeons of Great Britain within the past quarter of a century; yet there is a manifest reluctance to accept the views and experience of American surgeons in matters that have been clearly demonstrated to be safe and efficacious in practice within the last decade. It is reckoned as conservatism to be slow in adopting new measures, yet this has been carried to a point by English surgeons that amounts to obstinacy, and if not growing out of prejudice must be attributed to stupidity.

My space does not admit of details, but those familiar with the progress of surgery in this country will recall the facts which warrant this allegation as to some prominent members of the profession in England. Yet, all are not so wedded to the regime of the past as to refuse their sanction to the improvements of the present day, and there are enlightened representatives of progress in England and Scotland who keep pace with every new development in surgery. The precipitation which sometimes marks the zeal and energy of our people needs to be kept in check, when applied to medical and surgical researches; and perhaps the indisposition on the part of our neighbors to accept results without abundant proofs may in the end operate favorably, in leading those amongst us who investigate and experiment in the domain of surgery to look well to facts, in corroboration of theory or in support of practice, which may be recommended for the adoption of others.

The conservative character of British surgery in former days found a counterpart in the rather aggressive practice of French surgeons. Commencing with Ambrose Pare, Larrey, Dupuytren, Velpeau, Lisfranc, Nelaton, Civiale, and others of the initiative type of operators, the fame of the French capital was at one time above all other surgical centers, and yet in these later years the reputation of French surgeons has not kept pace with those of Germany and Austria. Billroth, Scroeder, Esmarch, Oldshausen, Volkman, Hegar, Simon, and Virchow have taken precedence in the competition for public preferment, and the victory of Prussia over France in 1870 seems to have been followed by the triumph of her surgeons in the operative field as well as in the pathological department.

Outside of the accidental importance given to Italy by the renown of Porro (from his substitution of ablations of the womb for the Cæsarean section in cases not admitting of pelvic delivery), there has not been any marked advances in that region. It would seem as if the Latin race had fallen behind the Teutonic descendants in the great practical developments of surgery during the last quarter of a century ; and while it is not within my province to discuss morals or politics, there may be a lesson drawn from the institutions of these countries which may throw some light upon their respective relations to the progress of the age.

There was in former days an impression that no one could be thoroughly trained for the duties of the surgeon without going abroad, and even in these latter days many seek *eclat* by visiting Paris, Vienna, Brussels, or Berlin who do not understand either the French or German language, and, as a consequence, receive little benefit by tarrying for a few months amidst the throng around the clinical professor whose fame is expected to recommend all who attend his lectures. Those who have money to spend and the time to dispose of, for attendance upon European teachers, during a period which may enable them to profit by their instruction, will most assuredly learn something of value ; but a flying visit, such as most of those seeking fame have made, has no real advantages for the student or for the practitioner. If our aspirants for distinction would turn to account a trip abroad, the best field for real improvement is the city of Edinburgh, and next to this comes the magnificent city of London, both of which afford ample facilities for clinical instruction upon a more utilitarian basis than in any other European cities. The prime consideration, however, is that the language of the teachers is understood so as to reap the advantages of a short sojourn in either of these English-speaking places, and the prestige of Bryant, McCormac, Lister, Sir Henry Thompson, Sir Spencer Wells, Sir James Paget, Lawson Tait, with the towering renown of Keith, ought to suffice for the ambition of all students or practitioners who look to the outside world for improvements in surgery.

But, thanks to the progressive spirit of the people in the United States, there is no sort of excuse for leaving this country to be most thoroughly fitted for every department of duty in the medical and surgical fields of service. What has not been accomplished in operative precedures and what cannot be attained by American surgeons is not likely to be realized in the civilized world, looking to practical results. Our race is pre-eminently initiatory and progressive, not only in industrial enterprises, but in philosophy, science and art; and some of the most important advances in surgery have had their origin in the inventive genius of the members of the profession in the United States.

We cannot cope with the French and Germans in abstract speculations, and in patient experimenting, which occupy a series of years to reach a conclusion, but in all that calls for a high order of intellect in investigation, and skillful application of means to attain any desirable object, the energy of those engaged in seeking practical results amongst us has not been equalled elsewhere.

I hereby claim before the medical world the first place for the American Surgeon, and defy England, France, Belgium, Austria, Italy, and Germany to produce men whose qualifications can be compared with our most skillful operators; and I challenge them to present an equal number, having like excellence with the average class of practitioners throughout the United States who have devoted their attention to surgery.

Lest it might appear an invidious distinction to name the most prominent surgeons in this country, where so many are entitled to high consideration, I would simply refer to Sims and Gross, who fell with their armor still girded for the fight, as types of American surgeons to whom those abroad as well as at home may be pointed with pride. Let us all emulate their example, and, proceeding with the conviction that whatever man has done man may do, "press toward the mark for the high calling" in the medical profession.

"The history of Surgery during the last few years," as well said by another, "presents us with a series of triumphs startling in their magnificence;" and "the authors of these great advances may well stand astounded before the results which they themselves have achieved."

We are no longer confined to the regime of the Past; and while it behooves us to hold fast to all the improvements which have been made, each member of the profession is a pioneer to make new explorations and open up new channels for advancement. Let no one think that progress is effected without labor, and patient industry. What is apparently accidental in the great discoveries of the age has really nothing haphazard in the steps by which the end is accomplished, but is the legitimate outgrowth of gradual development, and the result of painstaking investigation.

With cautious boldness our researches should be pushed up, the inclined plane of facts, going from the known to the unknown until we reach the platform of real Progress in Science.

